# The Use of Tuberculin

**Published:** 1901-07-27

**Authors:** 


### The Use of Tuberculin.

In opening the discussion, on Wednesday, on the therapeutic and diagnostic value of tuberculin in human tuberculosis, Dr. G. A. Heron said that with much enthusiasm and with high hopes of its future usefulness tuberculin was received in the autumn of 1890; but before the end of the following spring the remedy had to a very great extent fallen into disuse, and was damned as loudly as it had been welcomed. The outcry against its use was no doubt largely due to the statement made by Virchow to the effect that the use of tuberculin was attended with danger, the action of this preparation being in some cases to rouse dormant tuberculosis to fresh activity. At any rate its reputation was unmade as quickly as it had been made. Dr. Heron then entered into details regarding the 57 cases which have been treated by him at the Victoria Park Hospital since 1890, of which 51 were examples of tuberculosis of the lungs and 6 of lupus vulgaris. At the end of the year 1900, 17 of the 51 cases of lung disease had been lost sight of, but 16 of the remaining 34 were then known to be well and earning their living. Since March 1897 Dr. Heron has used only the new tuberculin. During that year ten cases were so treated in hospital. Two of these cases died — one was practically dying when the tuberculin was given — but the other eight (seven cases of tuberculosis of lung and one of lupus) all did well and left the hospital, urging as their sole reason for so doing their fitness for work and their desire to resume work. Three years later, *i.e.* in December 1900, two of them were dead; three were well and were supporting themselves by their work; three were lost sight of; and one, after remaining well till lately, had returned to hospital a few weeks previously because of a recurrence. In regard to the question of danger Dr. Heron holds that it is worse than useless to treat with tuberculin cases in which tuberculosis is complicated with other inflammation, but that in other appropriate cases tuberculin is at least as safe to the patient as any other potent drug. It does, however, involve some risk, and he related a case in point.

What, then, is the diagnostic value of tuberculin? That it produces its characteristic reaction wherever tuberculosis is present Dr. Heron thinks there can be no doubt. That it rarely fails to react where there is tuberculosis is so true that cases in which failure is recorded may safely be neglected. In his own experience of this use of it he has never seen any evil consequences follow its administration. As an illustration of the practical usefulness of tuberculin as a test in cases always difficult and often impossible of diagnosis by ordinary methods, he refers to the excellent work done in this direction by Dr. Eric France, of the London County Asylum, Claybury. His object was to ascertain, with certainty, who among the insane inmates of the asylum had tuberculosis. For this purpose he tested 55 of his patients with tuberculin. Characteristic reactions occurred in 45 of these cases. Thirty-four of them eventually died, and 29 of these 34 were submitted to post-mortem examination, with the result that, as Dr. France says, "Active tubercle was found in every case." Ten of the 55 patients did not react. Five of those died, and post-mortem, says Dr. France, "No trace of tubercle found in any; five still alive and healthy." Dr. Heron says that he feels sure the day is not distant when the discovery of tuberculin will be ranked among the most valued of the many gifts mankind already owes to Robert Koch.

